# New amino acid substitution matrix brings sequence alignments into agreement with structure matches

**DOI:** 10.1002/prot.26050

**Published:** 2021-02-02

**Authors:** Kejue Jia, Robert L Jernigan

**Affiliations:** Roy J. Carver Department of Biochemistry, Biophysics and Molecular Biology, Iowa State University, Ames, Iowa

**Keywords:** amino acid similarities, amino acid substitution matrix, interdependent amino acid substitutions, protein sequence matching, protein structure matching

## Abstract

Protein sequence matching presently fails to identify many structures that are highly similar, even when they are known to have the same function. The high packing densities in globular proteins lead to interdependent substitutions, which have not previously been considered for amino acid similarities. At present, sequence matching compares sequences based only upon the similarities of single amino acids, ignoring the fact that in densely packed protein, there are additional conservative substitutions representing exchanges between two interacting amino acids, such as a small-large pair changing to a large-small pair substitutions that are not individually so conservative. Here we show that including information for such pairs of substitutions yields improved sequence matches, and that these yield significant gains in the agreements between sequence alignments and structure matches of the same protein pair. The result shows sequence segments matched where structure segments are aligned. There are gains for all 2002 collected cases where the sequence alignments that were not previously congruent with the structure matches. Our results also demonstrate a significant gain in detecting homology for “twilight zone” protein sequences. The amino acid substitution metrics derived have many other potential applications, for annotations, protein design, mutagenesis design, and empirical potential derivation.

## 1 | INTRODUCTION

The rapid advances in genome sequencing and structural biology provide an opportunity to improve the understanding of the relationship between protein sequence and structure.^1^ Sequence matching is extremely important since it is by far the most commonly used computation in biology. The results from sequence matching serve as fundamental data for many studies.^2–13^ When utilizing the massive data of protein sequences, the combination of Basic Local Alignment Search Tool (BLAST)^14^ with amino acid substitution matrices is powerfully informative.

There is a long history of the development of a wide variety of substitution matrices. The point accepted mutation (PAM) matrices were the first set of substitution matrices developed by Margaret Dayhoff are based on 1572 point mutations in the phylogenetic trees of 71 families of closely related proteins. The blocks substitution matrix (BLOSUM) family of substitution matrices^15^ developed in 1992 are the most commonly used still, in part because of s use in BLAST at NCBI. The BLOSUM62 matrix is the default matrix for protein sequence matching there and remains the standard substitution matrix in protein sequence database searches and sequence matching. Miyazawa and Jernigan^16^ introduced another type of substitution matrix based on the empirical amino acid contact frequencies. Muller et al^17^ developed the variable time maximum likelihood (VTML) substitution matrices based on divergent alignments for identifying distantly related protein sequences. More specific substitution matrices were developed for different families of proteins^18^ or different types of structures.^19^ Yamada and Tomii^20^ reported a matrix based on principal component analysis and the variabilities across existing substitution matrices. Several reports have utilized sequence neighbors and sequence triplets^21,22^ to develop a pairwise or three-way dependent matrix, but these are only for nearest neighbors along a sequence. Other substitution matrices^23–28^ include some that have focused on the details of the matching method itself. None of the above mentioned approaches account for the interdependences of substitutions at longer separations in sequence.

People have also used structure information to derive substitution matrices. A straightforward way is to use protein structure alignment to extract the amino acid substitution information, based on the nearest residues in the two proteins in the match. Prlic et al derived substitution matrices from a set of protein structure pairs with high structural similarity but low sequence identity.^29^ The Johnson and Overington matrix also considered regions in the structure alignment, where the gaps occur.^30^ Blake and Cohen extracted substitutions from the superposition of protein structures to target the “twilight zone” (protein sequences with identity lower than 20%) in protein sequence matching.^31^ Teodorescu generated substitution matrices by combining the energy information from protein structures with substitution matrices derived from sequence data.^32^ However, only the BLOSUM substitution matrices have come into common e due to their limited improvements. Our own group recently explored the approach of developing different substitution matrices for different structure families but obtained relatively small gains.^33^ In a predecessor to this paper, we developed a new substitution matrix that combined BLOSUM62 matrix with the correlated pair information from multiple sequence alignments (MSAs)^34^ to show in a proof of principle that this can bring structure matches and sequence matches into agreement. That substitution matrix permits too many substitutions and was found to give false positives in homolog detection. The major challenge of incorporating structural information into a substitution matrix is to resolve the conflicting substitution information extracted from structures and sequences.

Studies from our group have developed knowledge-based potentials derived by mining residue contact information in protein structures that have proven to be the most successful type of potentials for assessing the quality of modeled protein structures at critical assessment of protein structure prediction (CASP).^35–37^ From this, we can hypothesize that the important interactions are those between neighboring residues. Globular proteins are tightly packed, almost at the density the most densely packed spheres.^38,39^ The packing density is also related in detail to sequence entropies and amino acid conservation^40,41^ because of the free volume around a given amino acid. In a densely packed environment, it is possible that these interdependences extend over long distances. One mutation may trigger a chain reaction that leads to a requirement for multiple coordinated mutations at its successive neighboring positions. The simplest of these dependences should be manifested in be a pair of coevolving positions. At two physical adjacent positions in a structure, a large-small pair of amino acids might be substituted with a small-large pair without disrupting the structure, or a charged (+−) pair might be substituted with a (−+) pair. These types of compensatory mutations can lead directly to substitutions at a single position that would not be considered to be so similar, but at the same time are maintaining the protein stability with closely similar free energies between the substituted structures. Since spatially close residues are often evolutionarily correlated, this means that coevolving residues has passed through correlated mutations in the history of evolution, and this is the underlying reason why it’s possible to make a connection between sequence and structure information by using these types of correlations.

The big data of protein sequences contain important information about correlated mutations. Such correlations can be detected by coevolution analysis methods. Mutual information based on the association of information entropy between a pair of residue positions is being widely and successfully used as a straightforward way to predict coevolved residues in structures from the apparent correlations in a sequence multiple alignment. The results are confounded by indirect correlations through intermediates.^42,43^ Dunn et al added in an average product correction to mutual information to remove the background correlation signals from random noise and phylogenetic components.^44^ Direct coupling analysis (DCA)^45,46^ was used to extract the direct correlations to predict the residue pairs in immediate spatial contact. To achieve a similar goal, Jones et al introduced protein sparse inverse of covariance, a precision matrix based approach to filter indirect correlations.^47^

**FIGURE 1:**
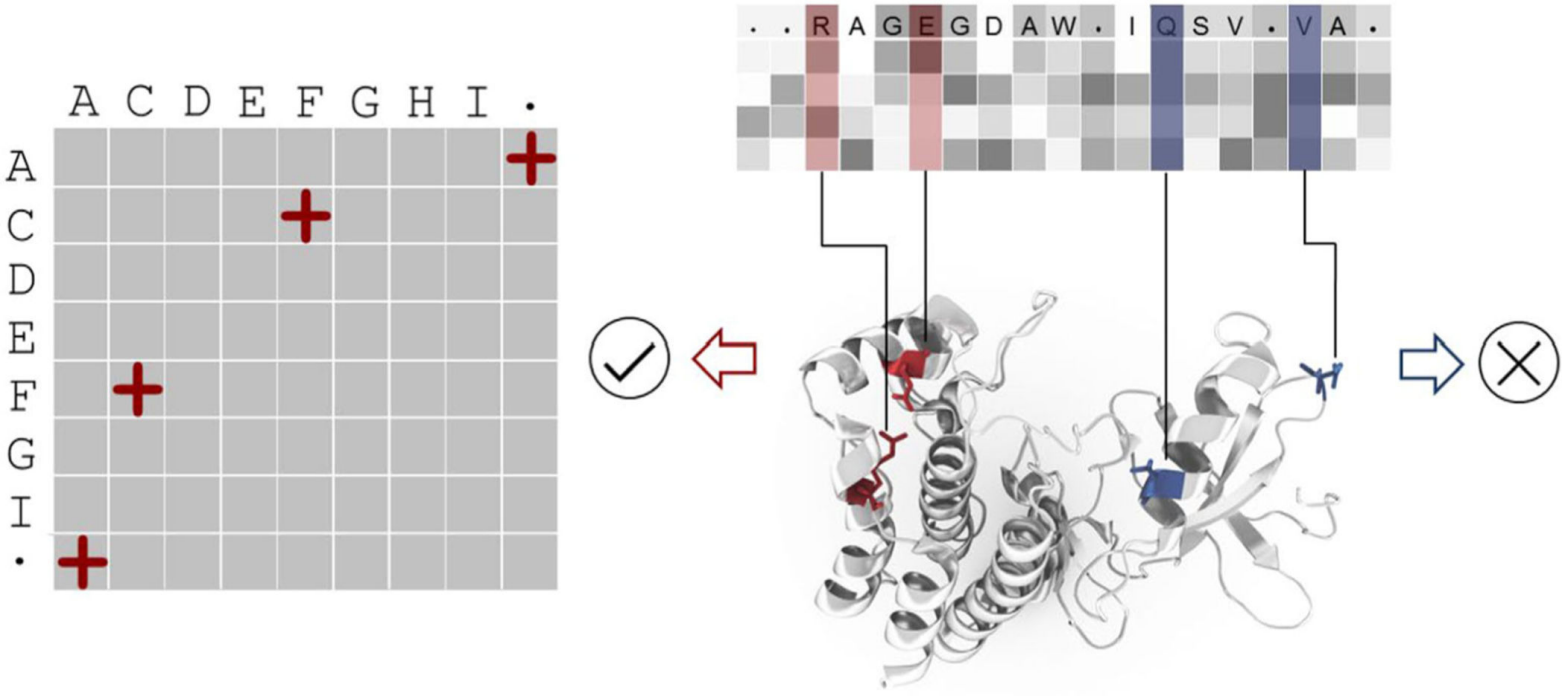
Derivation of the ProtSub amino acid substitution matrix. Pairs of residue positions having significant correlations in the multiple sequence alignments are filtered to retain only those correlated pairs in close contact (red) and discarding others (blue). 2320 Pfam domains were used to derive the sequence correlations. The resulting ProtSub substitution matrix is based on the cumulative information from all of these structural domains (see §4 in Supporting information for the list of domains whose multiple sequence alignments are used, together with the representative structures)

In the present study, we develop an approach to derive a new substitution matrix from a large set of sequence data that accounts for these interdependent substitutions and is intrinsically reflecting the structure information. The approach is shown in Figure 1. We first calculate the evolutionary correlation among pairs of positions in a MSA for a given protein family. Then filter to retain those pairs that have significant correlation with the additional requirement that they should be spatially close in the corresponding protein structure. Finally, the substitution matrix is derived from the interdependent amino acid substitutions contained in those pairs. Our results show that our new matrix is able to make congruent the sequence alignments and the structure matches. It also yields significant gains in identifying structural homologs for “twilight zone” sequences. We call this new amino acid substitution matrix “ProtSub” for Protein Substitutions.

## 2 | MATERIALS AND METHODS

### 2.1 | Datasets

One of the biggest challenges is to select a training dataset that covers as many protein families as possible and at the same time contains high-quality MSAs, that also have at least one representative protein structure for each family. A reliable coevolution correlation evaluation requires a large number of well aligned, diverse sequences. We chose to use the Pfam-A database^48^ because it is the largest protein domain family database that contains high-quality MSAs. The accuracy of Pfam MSAs is better because of their human curation. The selection of the training dataset is based on a balance among three criteria: (a). the coverage of protein families; (b). the number of MSAs that are suitable for coevolution calculation; (c) the quality of the representative structures. To test the ProtSub matrix, we use datasets from the (Class, Architecture, Topology and Homology (CATH)^49^ database and ASTRAL in structural classification of proteins — extended (SCOPe) database^50^ and follow the same testing schemes widely used in other studies. Both datasets have served as the “gold” standard for homolog detection test from previous studies. The testing dataset from the CATH database contains 4184 homolog families with a total of 8901 protein sequences. The testing dataset from the ASTRAL database contains 1454 homolog families and a total of 4066 sequences in the ASTRAL20 database. These two datasets ensure extensive coverage of homolog families and folding topologies.

### 2.2 | Extract coevolution correlations from multiple sequence alignments

In this study, we use 2320 Pfam-A MSAs to extract the coevolution correlations. Each family contains at least 1000 sequences and has at least one representative of an experimentally determined structure. In each MSA, only “ungapped” positions are considered in the calculation. Here an “ungapped” position is defined as a position containing fewer than 10% of gaps. In this study, we use mutual information to extract the coevolution correlations. Methods like DCA that predict residue contacts may lead to a biased sampling of interdependent substitutions. The transitive correlations resulting from mutual information also contain important information of interdependent substitutions, which should be considered in the process of sequence matching. The mutual information between two positions in a MSA is defined as:

(1) $$I_{i,j}=\sum_{A_{i},B_{j}}^{q}f\left(A_{i},B_{j}\right)\log\frac{f\left(A_{i},B_{j}\right)}{f\left(A_{i}\right)f\left(B_{j}\right)},$$

where *q* equals to 21, is the number of amino acid types and counts gaps as a 21st type of amino acid. While the number of sequence in the MSA data is sufficiently large, we use frequencies to approximate the probabilities. *f*(*A*_*i*_) is the frequency of a single amino acid type *A* observed at position *i*. *f*(*A*_*i*_, *B*_*j*_) is the joint frequency for observing the co-occurrence of two amino acid types *A* and *B* at positions *i* and *j*. Both single and pair frequencies are weighted by the redundancy of the sequences in the MSA (see §1 for details about weight calculation). We calculate *z*-scores for all the mutual information based on the approach described in reference^51^ and use a *z*-score threshold of 3.0 to define the significantly correlated pairs (See §2 Figure S1).

### 2.3 | Selecting correlated positions with observed proximate residues

False positive may be included when evaluating coevolution correlations. With all of the structure information available in the training dataset, we can use observed residue proximity to remove a significant number of these false positives. Here we limit the selected correlated position pairs to residue pairs that are structurally proximate. The tip atoms which are the heavy atoms on the terminal ends of each amino acid side chain (C-alpha for Glycine) have previously been used by Liang’s group^52,53^ to achieve higher specificities in empirical potential functions and for characterizing binding sites. In this study, proximate residues are defined by tip atoms from different residues that fall within a distance of 4.5 Å. Using tip atoms helps identify groups of proximate residues with large sequence separations that are most likely to be interacting.

### 2.4 | Derive a new matrix from contextual substitutions

The elements in an amino acid substitution matrix are substitution scores. A substitution score describes the tendency of one type of amino acid being substituted, or not, by another type of amino acid. The result of the coevolution calculation indicates that coevolution correlation is ubiquitous throughout every protein family. Most amino acid mutations do not occur independently, but depend on their structural context. By extracting substitution frequencies from correlated positions selected from the previous step, we embed the dependence information of substitutions into a new set of substitution scores. Similar to the original BLOSUM series of matrices, we also derive substitution scores using log-odds ratio:

(2) $$s(A,B)=\lambda \log\frac{f(A,B)}{f(A)f(B)},$$

where *f*(*A*, *B*) is the joint frequencies and *f*(*A*)*f*(*B*) are the expected single amino acid type frequencies. *λ* is a scalar factor, optimized to give the best homolog detection performance based on the training dataset (2320 Pfam domains). A semi-heuristic strategy is used here to optimize *λ*. First, a coarse set of values over a broad range of *λ* is investigated and then refined over a smaller interval, iteratively. The initial iteration scans through a range of values between 1.0 and 5.0 with a large interval of 0.5. The procedure stops when the stepping interval is less than 0.1. The outcome is to assign *λ* to be 2.4 in the final ProtSub matrix.

## 3 | RESULTS

**FIGURE 2:**
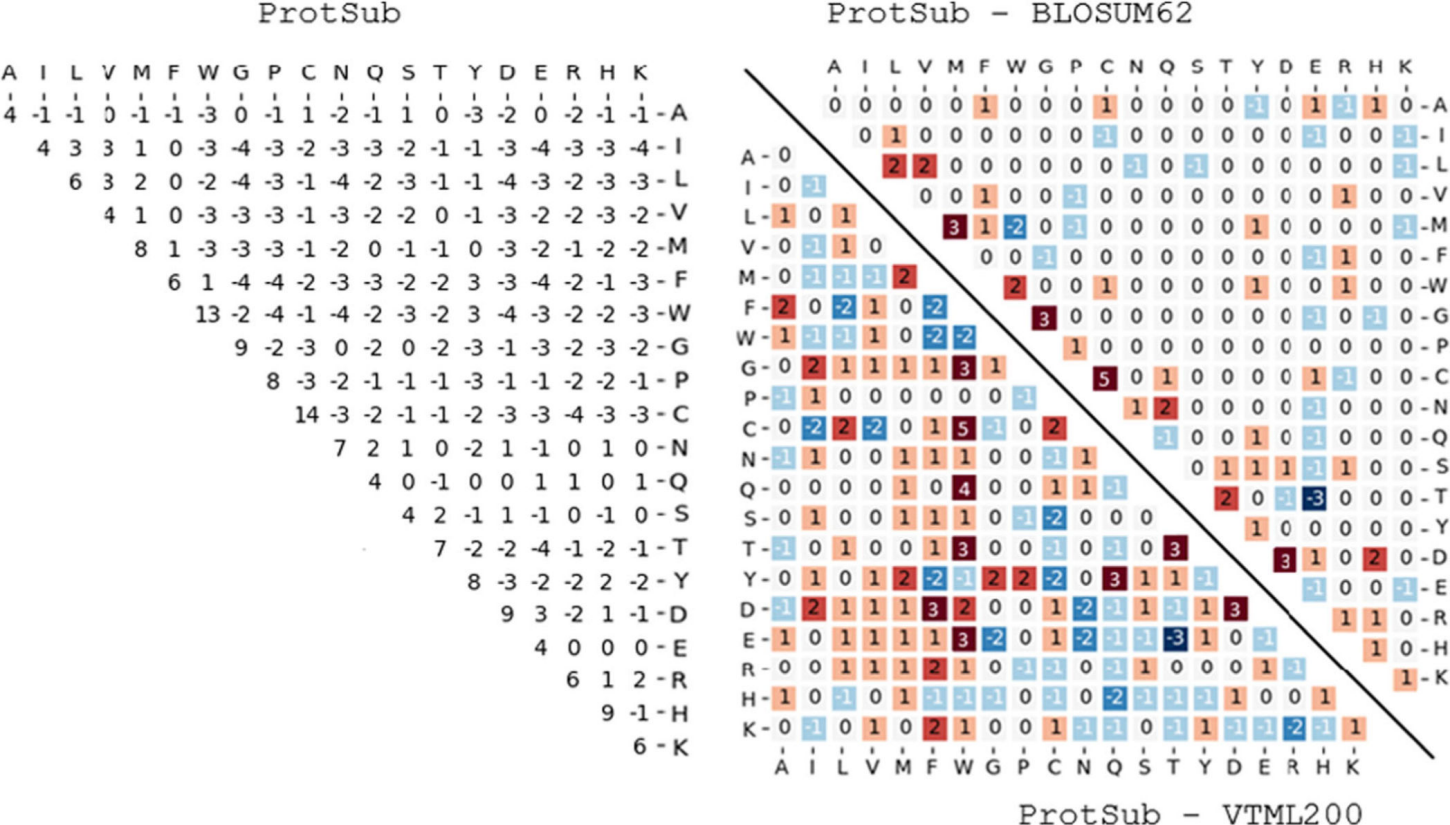
The ProtSub matrix and the comparison of amino acid substitution matrices between ProtSub and BLOSUM62, and between ProtSun and VTML200. The panel on the left shows the 210 nonsymmetrical elements of the ProbSub substitution matrix. The upper triangle of the panel on the right shows the differences between ProbSub and BLOSUM62 and the lower triangle shows the differences between ProtSub and VTML200. Increased scores are shown in red and decreased scores are in blue. A total of 63 of the 210 scores are changed (38 increases, 25 decreases) from BLOSUM62. A total of 139 of the 210 scores are changed (59 increases, 80 decreases) from VTML200

We compare ProtSub with two substitution matrices, BLOSUM and VTML200, which are reported to have the best performance in homolog detection from previous studies.^27^ Comparisons of the new substitution matrix for ProtSub with BLOSUM62, and VTML200 are shown in Figure 2. These differences are calculated by individually subtracting the corresponding elements in BLOSUM62 and VTML200 from the ProtSub matrix. Overall, ProtSub agrees more closely with BLOSUM62 than with VTML200. When compared with BLOSUM62, there are 63 out of the 210 elements changed. The major differences are on diagonal, instead of W being the most conserved, C is now the most conserved amino acid in the new ProtSub substitution matrix. It is difficult, however, to draw any particular conclusion about cysteines. The large score for cysteine in the matrix originates from its relatively low frequency of occurrence in the training dataset. The log-odd ratio calculation means that a rarer amino acid tends to have a larger diagonal score (conservation or self-substitution). In a MSA, conserved disulfides can mean higher conservation levels, which lead to low coevolution correlations. The criteria for selecting coevolved residues filter them out. In this case, those non-conserved cysteines considered in the matrix derivation appear to be relatively rarer than other types of amino acids. ProtSub permits more substitution among hydrophobic amino acids (L, V, M, F). There are also many small deviations scattered among the polar amino acids. Larger differences are seen between ProtSub and VTML200. There are 139 elements changed in total. In this figure, there are more discernible colored areas roughly separating matrix into two red and blue regions. The red region shows that ProtSub allows more substitutions between hydrophobic and polar amino acids. In contrast, the blue region stands for a slight trend of reduced values among the polar amino acids. Overall, the range of scores in ProtSub lies between BLOSUM62 and VTML200. In the BLOSUM62 comparison, there are 41 positive scores, which sum up to 148. There are 144 negative scores summing up to −303. Whereas ProtSub has 46 positive scores adding up to 183 and 140 negative scores which sum up to −312. The VTML200 comparison has much larger differences, with 53 positive scores summing up to 202, and 132 negative scores sum up to −369. The main reason for these differences is that probsub includes information about the correlated substitutions. Due to the compensatory effect, the amino acids at two positions may swap positions during mutation. This results in some substitutions that seem not so similar if only the substitutions at a single position are considered. Second, compared with BLOSUM62 and VTML200, ProtSub has a much larger training data set with longer alignment lengths, means that it is a more complete sample. Also, the longer sequences allow longer-range interdependent substitutions to be reflected in the substitution scores. Finally, as the scaling factor *λ* adjusts the magnitude of the overall score, some differences are also adjusted accordingly. There will be further discussion about in the discussion section.

### 3.1 | Better sequence alignments of structurally similar parts

**FIGURE 3:**
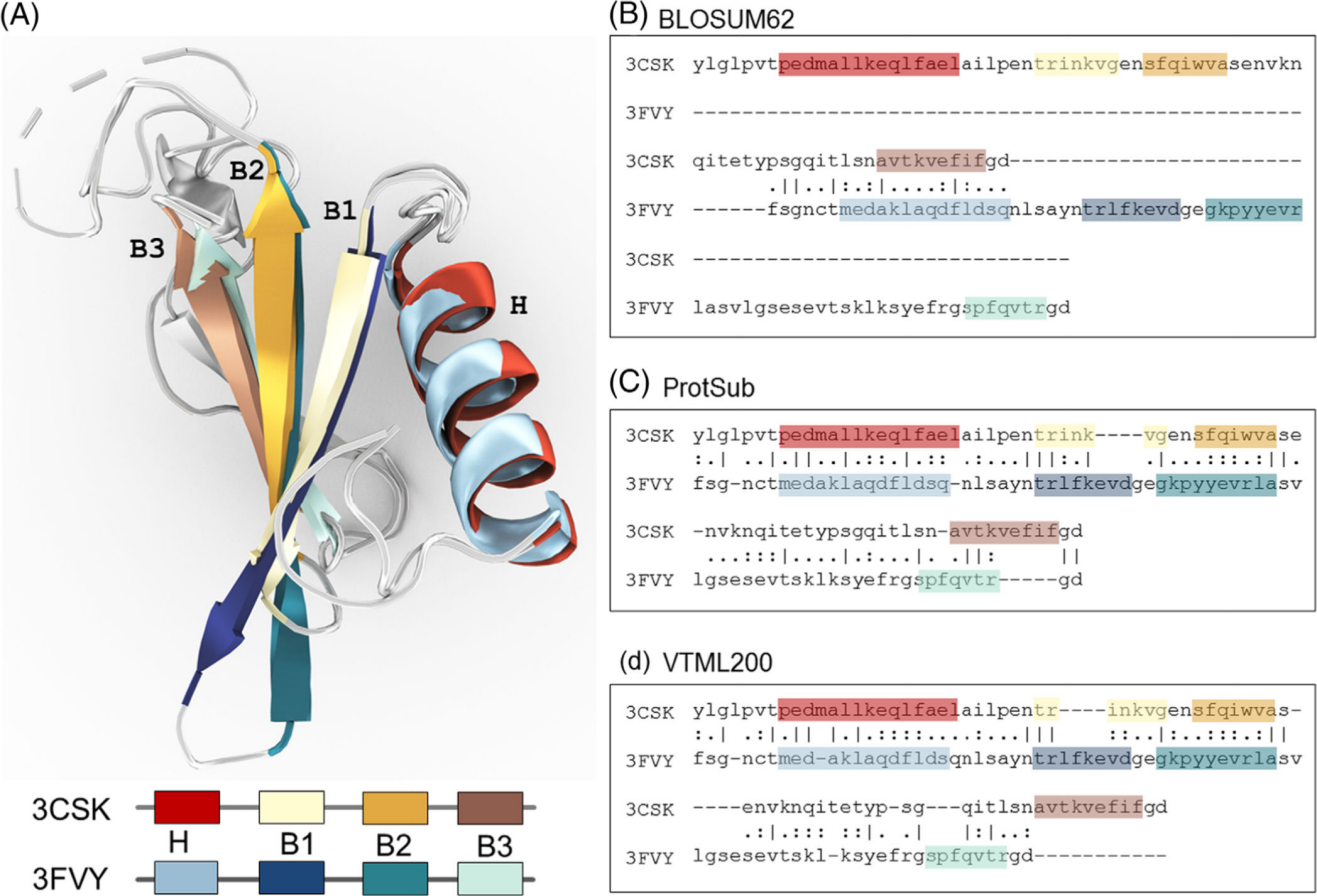
Sequence alignment with ProtSub is best in successfully matching the alignments of the specific structure segments, A. Structure match of protein structures PDB ID: 3CSK and 3FVY are both Dipeptidyl Peptidase III enzymes and one domain is considered here (3CSK, 184–259 and 3FVY, 172–250). The two protein structures are nearly identical. The structure alignment matches the one helix (H) and three β strands (B1, B2, B3). The paired colors in the key indicate which part of the structures should be aligned in the sequence alignment. B, the sequence alignment generated with BLOSUM62 does not appropriately match any of the structure features. The sequence alignments generated by ProtSub are shown in C, with excellent matches of the secondary structure elements. D, the match with VTML200 aligns H, B1, B2 similarly to ProtSub but provides no alignment for B3. ProtSub produces a sequence alignment strongly agreeing with the structure alignment. The root mean squared deviation (RMSD) based on the sequence alignments for ProtSub is 5.32 Å over 69 aligned amino acids. BLOSUM62 has 20 aligned amino acids with a RMSD of 20.17 Å and VTML200 has 62 aligned amino acids with a RMSD of 8.7 Å. The sequence alignments are generated using Needleman-Wunsch in the EMBOSS software, with the default gap penalty (10.5 for the gap open and 0.5 for the gap extension)

**FIGURE 4:**
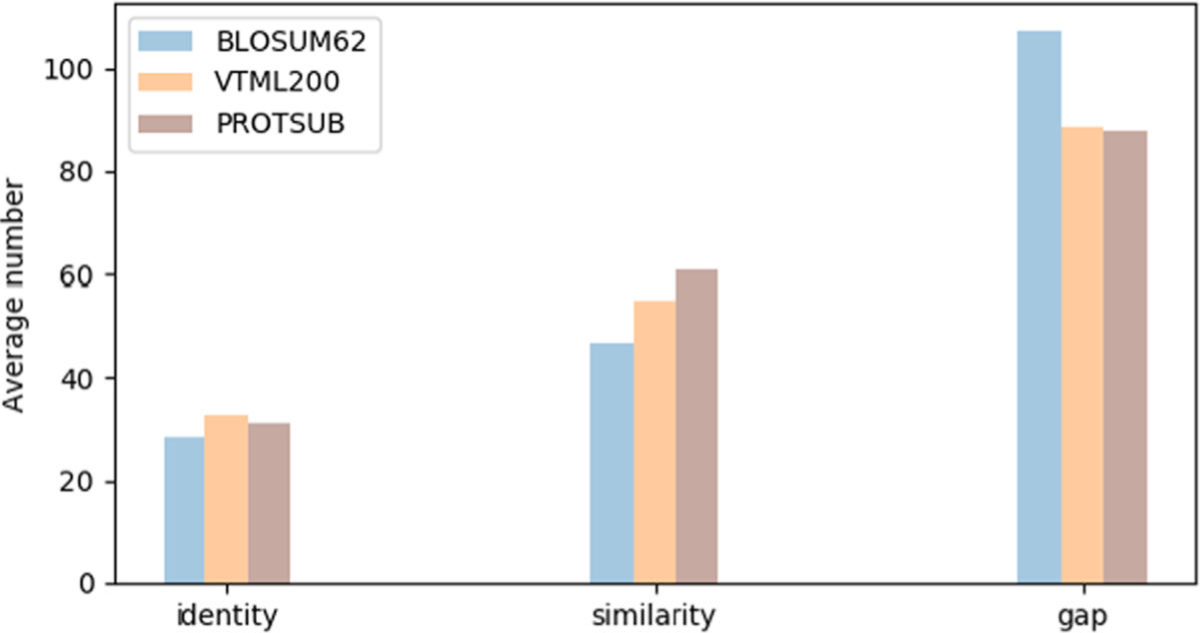
Performance of three substitution matrices. The average sequence identity and sequence similarity from the use of ProtSub, BLOSUM62, and VTML200 for 2002 sequence matches

In this study, we claim that using correlated substitutions integrates structural information into the new sequence substitution matrix. So the sequence alignments generated with this matrix should agree more closely with the results of structure alignments. There are a substantial number of known cases where two evolutionarily related proteins have almost identical folds but their sequences are not at present considered to be similar. We extract in total 2002 non-redundant pairs of sequences from the CATHS20 dataset that have this feature. (see §3 in Supporting information for the list of protein structures from the CATH database). We have made certain that: (a) there are at least two pairs of sequences selected from each homolog family; and (b) the selected sequences do not contain any Pfam domains used in deriving the new matrix. We use the global alignment procedure in the European molecular biology open software suite (EMBOSS) to align each pair of sequences^54,55^ and use template modeling align (TM-align)^56^ to generate the structure alignments. (Note: we also carried out the structure alignments with CEalign^57^ and found only minor differences in the overall results.) As a result, ProtSub achieves better agreements between the sequence alignment and the structure alignment for these cases. An example of an improved alignment in Figure 3 shows that the aligned sequence segments are positioned to agree with the structural alignment. The summary of sequence alignments for all 2002 cases under a default gap penalties (gap opening: 10, gap extension: 0.5) is shown in Figure 4 for the changes. The number of identities is slightly higher in comparison with the corresponding BLOSUM62 match, but slightly lower than with VTML200. The substitution scores for ProtSub are higher than for BLOSUM62 and VTML200. The number of gaps is reduced substantially over BLOSUM62 and only very slightly more than with VTML200.

**FIGURE 5:**
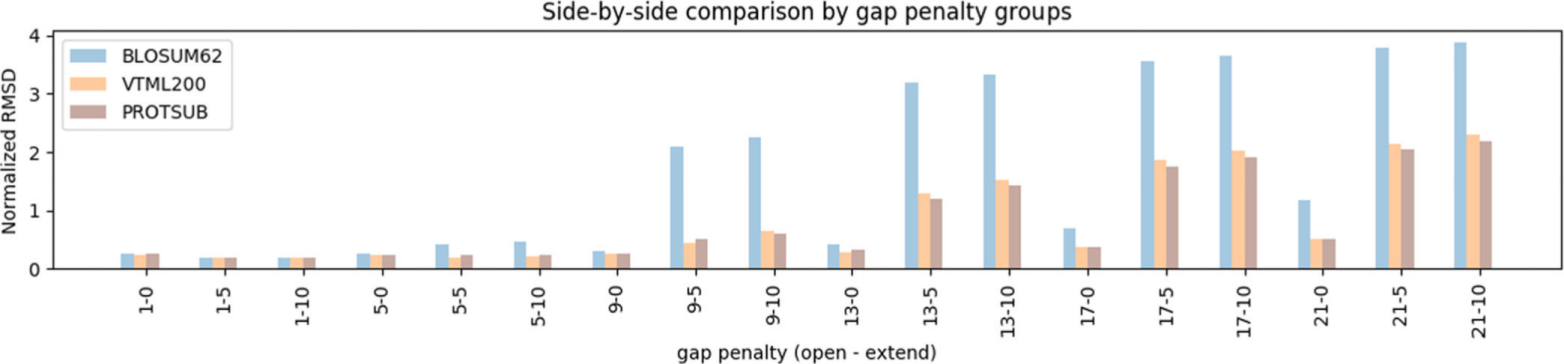
Normalized root mean squared deviation (RMSD) based on sequence alignments. For most of gap opening and extension combinations, the aligned segments from ProtSub have lower average RMSD than from BLOSUM62 and VTML200 in the structure alignments for the 2002 H-level cases

In order to measure the extent of agreement between a sequence alignment and a structure alignment, the root mean squared deviation (RMSD) is calculated from the structure alignment for residue pairs in the aligned sequence segments as specified in the sequence alignment. To obtain a comprehensive comparison, we iterate different gap penalties with a fixed interval to generate sequence alignments. Gap opening penalty ranges from 1 to 21 in steps of 1.0, gap extensions from 0 to 10 in steps of 5.0. The mean RMSD of all 2002 cases is calculated based on sequence alignments generated for different gap opening and gap extension combinations, as shown in Figure 5. The results clearly demonstrate that ProtSub matches the structurally similar parts better than BLOSUM62. ProtSub and VTML200 yield similar results when the gap penalties are low, but starting at a gap opening value of 13, and gap extension parameter of five, ProtSub does slightly outperform VTML200 for other higher gap penalties.

### 3.2 | ProtSub performs better for matching “twilight zone” homolog protein sequences

**FIGURE 6:**
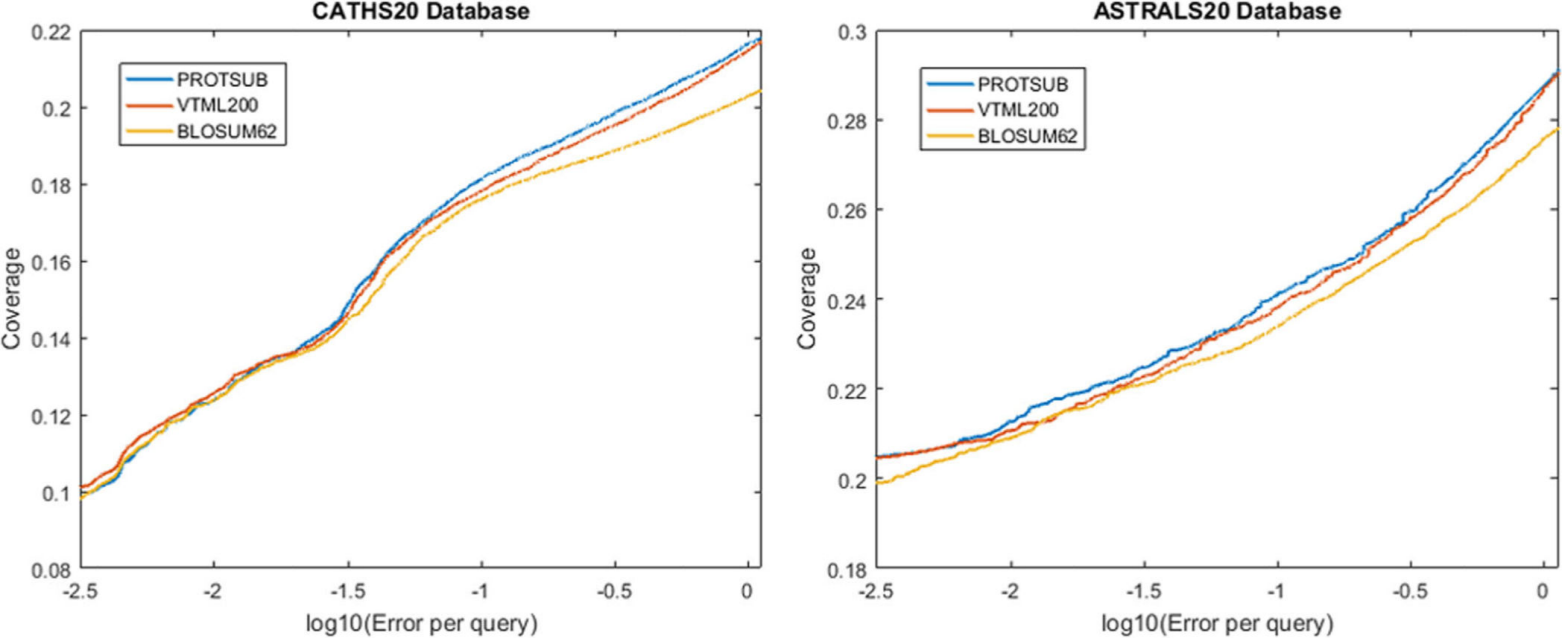
Coverage vs errors plots of the best-to-best comparison of homolog detection between Protsub and BLOSUM62, VTML200. The left panel shows the test results for the ASTRAL20 dataset and the right panel shows the results for the CATHS20 dataset. The ordinate is the coverage, which is defined as the fraction of true positive homologs that have scores above a certain threshold. The abscissa is the error rate, which is defined as the number of non-homologous sequences above the threshold divided by the number of queries on a log-scale

Sequence matching can successfully distinguish between pairs of protein sequences when the sequence identity is high. The results become unreliable, however, for matching “twilight zone” sequences (<20% sequence identity). To test the performance for homolog detection with the ProtSub matrix, we make the best-to-best comparison among the new ProtSub, BLOSUM62, and VTML200 matrices. Each sequence in the test dataset is queried against the whole dataset. Each query is also performed for all 11 build-in gap opening and extension combinations in BLAST: (2, 9), (2, 8), (2, 7), (2, 6), (1, 11), (1, 10), (1, 9), (2, 11), (2, 10), (1, 13), and (1, 12). Then the best result for each matrix is selected to make the comparison. To avoid the bias from testing on only a single dataset, we perform the same all-to-all homolog testing on both the datasets of ASTRALS20 from SCOPe and CATHS20 from CATH database. These are the two datasets with human curated classification information for “twilight zone” protein sequences, both having less than 20% sequence identity. The ASTRAL20 dataset is a subset of the SCOPe database. This dataset has been commonly proposed as a “gold” standard for evaluating the performance of homology search methods. After removing sequences that contain Pfam domains used in the training dataset for developing the ProtSub matrix, the ASTRAL20 has in total 1454 homolog families and 4066 sequences with no greater than 20% identity to one another; The CATHS20 contains 4184 homolog families and 8901 protein sequences, which also have no more than 20% identity to one another. The results demonstrate that ProtSub outperforms for both BLOSUM62 and VTML200 in homolog detection on both datasets (Figure 6).

To evaluate the performance, we use coverage vs errors per query (CVE), a method developed by Price, et al.^58^ The CVE curves are similar to receiver operating characteristic curves, but present the results in a way that is directly interpretable and suitable for sequence analysis. The “coverage” on the Y-axis is defined as the fraction of true positive homologs that have scores above a certain threshold; this reflects the sensitivity of the matrix. On the X-axis, “errors per query” (EPQ) acts as an indicator of selectivity, which shows the number of non-homologous sequences above the threshold divided by the number of queries. The EPQ axis in a CVE plot is on a log scale to show performance over a wide error range. These homolog searches are performed in an all-to-all fashion, which takes each one of sequences in the dataset as a query sequence to search against all others. In order to generate the CVE curves, the results of all queries are sorted in the order of decreasing statistical significance (using e-values from BLAST). In this case, by traversing the sorted list from top to bottom, the coverage is accumulated by counting the true positive homologs. We test all possible gap opening and gap extension parameters (as used by BLAST) and find the optimal set of gap parameters for each matrix. The CVE curves shown in Figure 6 are best-to-best performance comparisons. The gap penalties for the ProtSub matrix that give the best performance are (2, 9) and (2, 6) for CATHS20 and ASTRAL20, respectively. For the CATHS20 dataset, the three substitution matrices perform similarly when the tolerance for errors is low. After the tolerance reaches around one error per 30 queries (−1.5 on the X-axis), ProtSub outperforms the other two matrices. VTML200 performs slightly better at the beginning and performs similar to ProtSub on the rest of the curve. For the ASTRAL20 dataset, ProtSub outperforms the others from around one error per 160 queries (−2.2 on the X-axis). For both test datasets, BLOSUM62 perform the worst. The advantage of ProtSub increases as the error tolerance increases.

## 4 | DISCUSSION

The combination of BLAST with BLOSUM62 has been the most commonly used procedure for matching sequences for many years. A comparative study showed that VTML200 gave the best detection performance in detecting homologs for sequences having low sequence similarity in comparison with the commonly used BLOSUM, PAM and other series of matrices.^27^ Since the BLOSUM62 and VTML200 derivations were based on a significantly smaller set of sequence alignments, there are gains from utilizing the present much larger sets of structure and sequence data. In addition, our results show gains over BLOSUM62 and VTML200 because of utilizing more diverse data: utilizing compensating substitutions for closely interacting residues and MSAs of larger domains. The major improvement in the performance of protein sequence matching derives substantially from incorporating structural information into the ProtSub matrix.

### 4.1 | Comparing the BLOSUM BLOCK dataset with Pfam multiple sequence alignments

The calculation of log-odds ratios in ProbSub is similar to BLOSUM62 but the training data that is used to derive amino acid substitutions is different. The BLOSUM series of matrices were derived from more than 2000 ungapped local alignments called blocks. Each block alignment is considered to be a conserved region in a protein family. Blocks are generated using the PROTOMAT tool.^15^ PROTOMAT is a two-step system that first extracts motifs from raw sequence data with the help of an amino acid substitution matrix and then generates block alignments using motifs. The block alignments that were used to generate BLOSUM matrices are refined iteratively, that is, a new substitution matrix is obtained by using the block alignment generated for the first time, then PROTOMAT uses this matrix to refine the block again, and finally uses the refined block to generate the final BLOSUM matrices.

In this study, we extract correlated mutations using Pfam-A MSAs. The Pfam-A database uses a Hidden Markov Model (HMM) based on a probabilistic modeling technique to construct the alignments for different protein domain families. For each protein domain family, a small-scale seed alignment is first generated from the raw protein sequence data. This seed alignment is curated by humans to increase the accuracy. The seed alignments are also compared with several existing databases including CATH,^49^ SCOPe,^50^ and evolutionary classification of protein domains (ECOD)^59^ as a quality control. Using the HMM profile extracted with the seed alignment, the final full alignments are generated by searching a large-scale protein sequence database, such as Uniprot.

The ungapped block method is robust and fast for the conserved regions of the protein sequences in the database. However, gaps may occur in conserved regions. The restriction of no gap not only puts a limitation on the true positive coverage but also can lead to truncation of domains. Thus, the major difference between block alignments and Pfam-A alignments is that block alignments include short conserved regions, while Pfam-A alignments provide complete domains. As a result, the correlated mutations are underestimated due to the truncation of domains in the short ungapped block alignments. The block alignments used to generate the matrix contain approximately 27 000 sequences. The data has exploded recently. Here, we use more than 3000 MSAs of Pfam-A domain families, and each has more than 1000 sequence available. Our sequence number for training is more than 124 million (12 438 029) in total. The blocks used by the Henikoffs to develop the BLOCK matrices were around 60 residues in length, and so these necessarily are omitting most of the relatively common longer-range effects.

### 4.2 | The extent of permitted substitutions in a substitution matrix affects test results

The test results are strongly affected by the amount of permitted substitutions permitted by a given substitution matrix, which reflects the probabilities in the training dataset. In the procedure of aligning sequences, adding log-odds scores can be thought of as multiplying the corresponding probabilities all together. As shown in Figure 2, the extent of permitted substitutions with ProtSub lies between that of BLOSUM62 and VTML200 and closer to BLOSUM62. In both tests, ProtSub performs best and the results are closer to VTML200 than to BLOSUM62. In part, it appears that VTML200 achieves its results by being too permissive in accepting substitutions.

### 4.3 | The scale of the scores affects the test results

**FIGURE 7:**
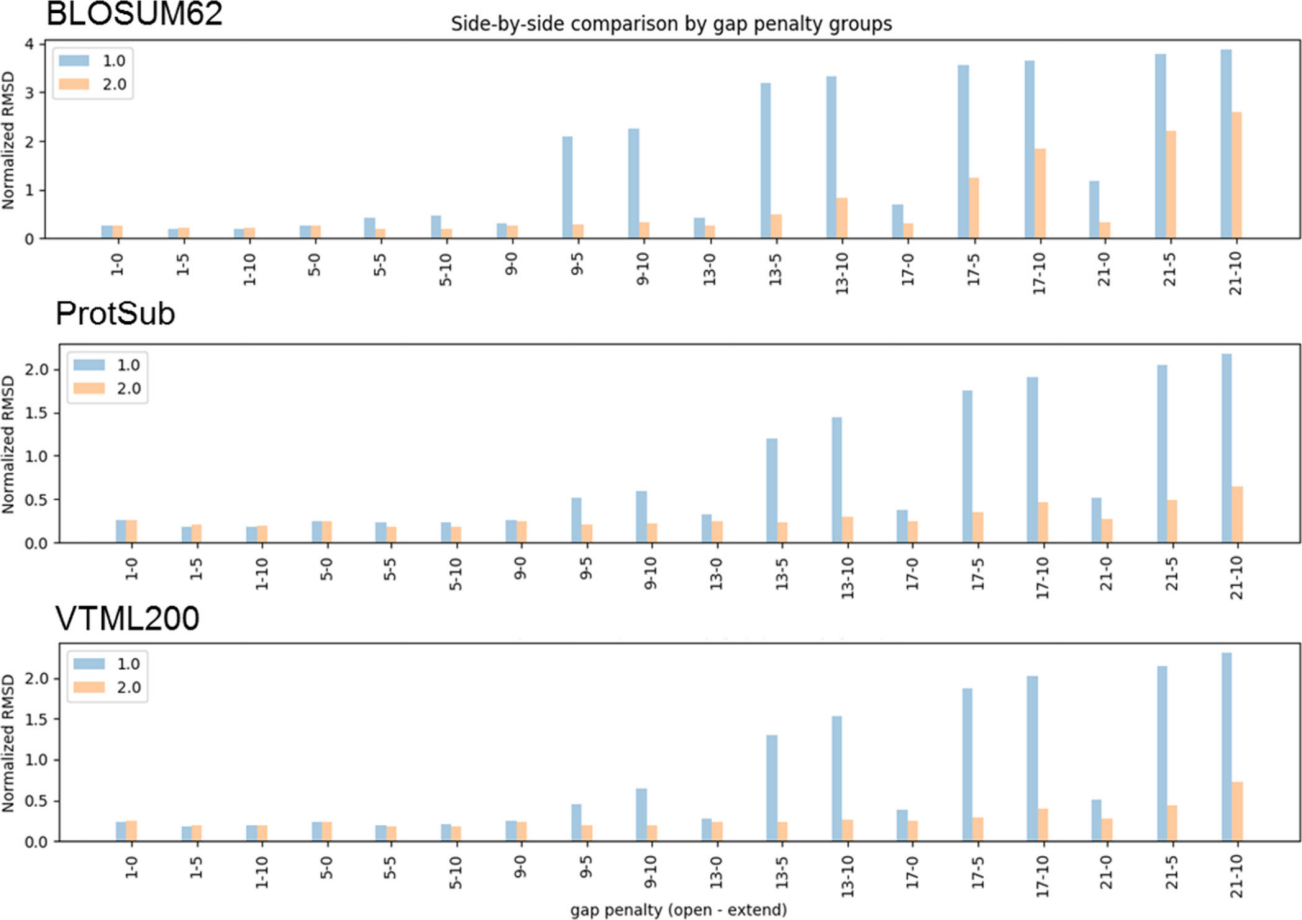
Comparison of the original matrix (1.0) with the scaled matrix (2.0) in the global alignment test. The scaled matrices are less sensitive to gap penalties than the original matrices

**FIGURE 8:**
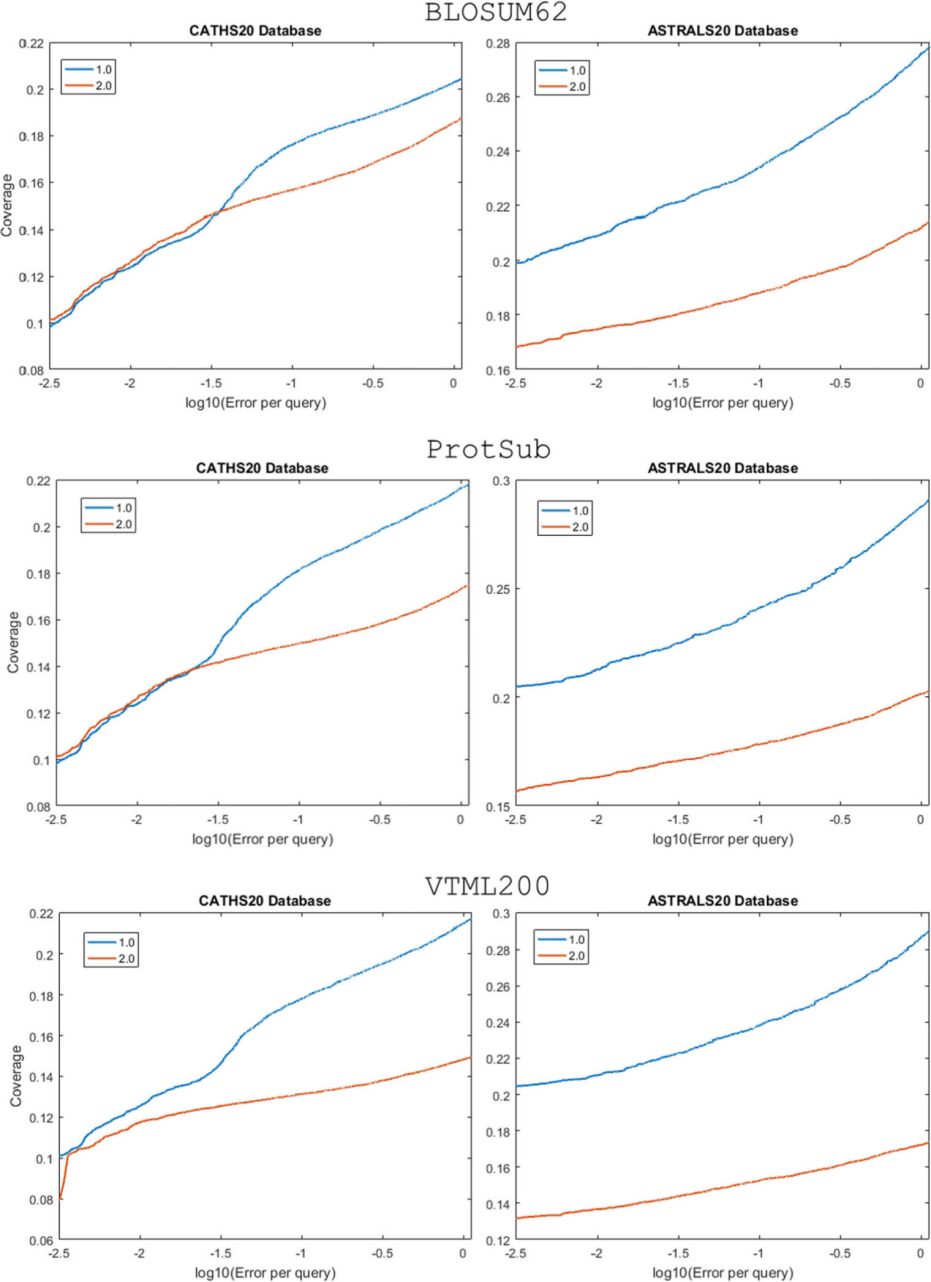
Comparison of the original matrix (1.0) and the scaled matrix (2.0) in the homolog detection test. For all three matrices (BLOSUM62, VTML200, ProtSub), scaled matrices perform worse than the original matrices

The scale of the scores in a substitution matrix can also affect its performance in both tests. Multiplying a substitution matrix by a constant changes the *λ* in Equation (2) but does not alter the matrix’s implicit target frequencies. Such a scaling corresponds to using a different implicit base for the logarithm. For global alignments, multiplying all scores by a fixed positive number has no effect on the relative scores of different ungapped alignments.^60^ However, when gaps are included, the scale of the scores does affect the final alignment. To test the scaling effect, we generated matrices that scale the original scores of the three substitution matrices (BLOSUM62, VTML200, and ProtSub) by scaling factors up to 2.0. Then we perform the same tests using those scaled matrices. As a result, we find that the larger the scale of the matrix, the more insensitive it is to gap penalties. In the global alignment test, matrices with a larger scale of scores do generate more compact alignments than the original matrices (Figure 7). However, in the homolog detection test, they yield significantly larger numbers of false positives than do the original matrices (Figure 8). It is consistent with the point made by Karlin and Altschul that scaling a substitution matrix will affect local alignments.^61^

## 5 | CONCLUSION

Applications of ProtSub can advance molecular, genomic, structural, and evolutionary biology. A few of its most important gains will be improved gene annotations such as in Figure 3, improved protein structure predictions, improved evaluations of the effects of mutations, and better tools for carrying out protein design. The ability to identifying broader sets of homologs can help researchers identify related proteins in different organisms.^62^ In the field of comparative genomics, it can also provide useful information for identifying functions of proteins across diverse species.

### DATA AND CODE AVAILABILITY

The Python scripts used for calculating the mutual information and deriving conditional substitution matrices are available on GitHub: https://github.com/jkjium/contactGroups; Pfam MSAs used in this study were taken from the Pfam database (version 31.0): https://pfam.xfam.org/. The list of Pfam Domains and the percentage of residue positions used for the final ProtSub matrix derivation are provided in the Supporting information §4 Table S2. The listed residue positions are given in Supporting information §5 with the surface accessibility calculated using NAccess tool^63^ and the secondary structure evaluated by d(DSSP) software.^64^

The “twilight zone” sequences and PDB structures used for sequence alignment comparisons are from CATH (CATHS20) database: https://www.cathdb.info. The global alignments are generated by using the Needleman–Wunsch method from EMBOSS https://www.ebi.ac.uk/Tools/emboss/. The structure alignments are generated with the TM-align tool. The list of PDB IDs used for structure matching is given in Table S1 and the list of Pfam IDs used is provided in Table S2. The new ProtSub substitution matrix is provided in EMBOSS format in the Supporting information §6.

## Supplementary Material

supinfo
